# Evaluation of the Combined Application of Ultrasound Imaging Techniques for Middle Cerebral Artery Stent Surveillance and Follow-Up Study

**DOI:** 10.1371/journal.pone.0079410

**Published:** 2013-11-13

**Authors:** Yu Wang, Jian Mei Chen, Xi Liu, Jia Wang, Li Hong Li, Jian Ping Deng, Yun You Duan

**Affiliations:** 1 Department of Ultrasound Diagnostics, Tangdu Hospital, Fourth Military Medical University, Xi'an, China; 2 Department of Neurosurgery, Tangdu Hospital, Fourth Military Medical University, Xi'an, China; University Medical Center (UMC) Utrecht, The Netherlands

## Abstract

**Objective:**

In recent years, cerebral artery stenting has become an effective method for the treatment of cerebral artery stenosis. However, methods for assessing efficacy and techniques for follow-up imaging still need to be developed. This study was designed to evaluate the application of transcranial color-coded sonography (TCCS) in assessing stenting of middle cerebral artery (MCA) stenosis. And, two new imaging techniques (vascular enhancement technology (VET) and 3-dimensional (3D) imaging) were tried out and evaluated.

**Method:**

We enrolled 43 patients with cerebral artery stenosis for vascular stent implantation. All patients were examined by ultrasonography and confirmed through digital subtraction angiography. The stenosis was imaged and blood flow parameters were analyzed before and after the procedure using TCCS. VET and 3D imaging model were used in part of the patients. Important postoperative hemodynamic changes were noted.

**Results:**

1) Adequate stent image was present in 41 out of 43 patients as detected by postoperative 2-dimensional imaging. Images lacking clarity were obtained in 2 patients. 2) The perioperative and postoperative (one week follow-up) instantaneous blood flow velocity at the site of stenosis was significantly decreased (P<0.05) when compared with preoperative levels. Differences between postoperative (one week follow-up) and preoperative blood flow velocity were significant (P<0.05). Differences in blood flow velocity at long-term follow-up (six months and two years) compared to one-week values were not statistically significant (P>0.05). 3) VET imaging visualizes the MCA lumen and stent morphology clearly. 3D ultrasound can be used for imaging of the stent shape as well as its inner surface.

**Conclusion:**

TCCD can be considered a quick and effective clinical detection method to evaluate the intracranial arterial hemodynamics changes before and after stenting treatment for MCA stenosis. New imaging technologies 3D and VET can achieve additional image information.

## Introduction

The high morbidity and mortality of ischemic strokes is a global healthcare concern. Intracranial atherosclerotic stenosis leads to a sharp decline in blood flow to brain tissue and is one of the important causes of ischemic strokes. The intracranial stenosis incidence is quite different due to the race, ethnic and region, and it is of importance in certain parts of the world such as some countries and regions including China. Past research implicates middle cerebral artery (MCA) stenosis as the most common site of symptomatic intracranial atherosclerosis [1]. The degree of narrowing at the site of stenosis is associated with new-onset ipsilateral vascular events. A hemodynamic mechanism is one of the main factors contributing to recurrent ischemic strokes in these patients [2]. In recent years, intravascular stent placement for treatment of carotid stenosis has been gradually improving. New techniques have emerged which permit intracranial vascular stenting [3]–[4]. In patients who are not responding to antithrombotic therapy or have recurrent symptomatic MCA stenoses, intravascular stenting has been the preferred preventative treatment and can significantly improve low levels of local perfusion due to MCA stenosis, thereby preventing ischemic strokes and significantly reducing the incidence of strokes [5]–[6]. However, intracranial stenting is feasible but that it is not substantiated by any randomized clinical trial, and, methods for intracranial detection of stent placement are still limited. While DSA is the gold standard for the diagnosis of intracranial arterial stenosis, it is nonetheless expensive, invasive, and cannot present hemodynamic changes. As a result, repeated DSA tests within a short time span are not preferred and therefore cannot be used for follow-up testing after stent placement.

Transcranial color-coded sonography (TCCS) provides real-time, dynamic morphological information through color or power Doppler flow imaging. Doppler wave spectrum and the measurement of blood flow parameters enable evaluation of hemodynamic changes and cerebral vasospasms [7]–[8]. In recent two decades, it is proved and accepted as an effective tool in intracranial vascular disease. However, there is seldom study of TCCS in intracranial stenting because the stenting procedure is not substantiated by any randomized clinical trial. Morreira et al. analyzed DSA/MRA and ultrasound imaging data of patients with neck and intracranial stents. Based on the results of the 2-year follow-up, a reliable determination of stent restenosis can be made based on blood flow velocity measurements established through ultrasound [9]. With the development of ultrasound imaging, some novel techniques such as like vascular enhancement technology (VET) and three-dimensional (3D) imaging are approved to be valuable in clinical diagnosis. 3D imaging can achieve more space information of hollow viscera organs than traditional two-dimensional imaging. The VET technique can provide clear images of the vessel lumen by “reverse enhancement” of micro-vascular display capabilities. The report of 3D and VET mode imaging on intracranial stenting is rare. We designed this study to evaluate the clinical application of ultrasound in the management of stent placement for MCA stenosis. We used traditional TCCS as a follow-up method to assess hemodynamic changes at various time points before and after the MCA stent placement, and, applied VET and 3D imaging to part of the patients to test whether could we get additional information.

## Materials and Methods

### Research subjects

Between July 2008 and July 2012, we enrolled 43 patients with cerebral artery stenosis at our institution consecutively. Thirty-two patients were male and 11 were female ranging in age between 32–69 years (mean age of 54.5±10.9 years). Patients with the clinical symptoms of cerebral artery stenosis included the following: sudden cerebral ischemia or limb weakness, paralysis, sensory disturbances, difficulties in producing language or hemianopia. By TCCS examination, only patients with a sufficient bone window and with acceleration of the MCA blood flow velocity were included into the study. In patients included in the study, ultrasound detected abnormally high rates of blood flow velocity at the point of the stenosis. The location of the stenosis was at the M1 segment of the MCA. All patients were examined and confirmed through digital subtraction angiography (DSA). We used the Gateway-Wingspan stent (Boston, United States), which is 2.5–3.5 mm in diameter and 15–20 mm in length.

#### Ethical approval of the study protocol

All participants (or their legal guardians) gave written informed consent according to the Declaration of Helsinki. The study was approved by the Human Subjects Review Committee of the Fourth Military Medicine University (Xi'an, China).

### Instruments and methods

The Siemens Antares and Acuson Sequoia512 color Doppler ultrasound system were used. The probe model was 4P1 or 4Vc1 with a frequency of 1.8– 2.0 MHz. The MCA probe depth was 120– 160 mm, and transcranial Doppler imaging settings were used.

For preoperative examinations, subjects were required to be sober and calm but were not required to undergo special preparations. During temporal window examinations, patients were positioned in the lateral position and the probe was placed above the zygomatic arch between the lateral orbital margin and the earflap. Then, the probe was moved to the front, middle or rear windows depending on the image display. In 2D ultrasound, the clear “heart-shaped” hypoechoic structure originating from the midbrain was used as a positioning marker. Color Doppler flow imaging (CDFI) or power Doppler functions of the instrument can be used to display the major blood vessels of the circle of Willis at the base of the brain. Individual blood vessels can be located using blood flow direction and vascular anatomy information obtained through CDFI. Adjustments were made to the color scale and gain to yield clear images. Red indicates blood flow toward the probe and blue indicates blood flow away from the probe. Through the temporal window, it is possible to observe the ipsilateral MCA, which is shown in red. Further assessments can be made on the even distribution of the blood flow stream and the presence of stenotic lesions. Spectral Doppler was then enabled and line sampling and color flow beam were obtained at an angle <60°. Samples were taken at vessel locations once every 0.3–0.5 cm. The spectral shape of blood flow as well as changes of the audio signal at each sampled location was monitored. The following blood flow parameters were measured: systolic peak flow rate (Vmax), end-diastolic flow rate (Vmin), time-averaged maximum flow rate (Vmean), and resistance index (RI).

2D ultrasound was used to determine stent location and morphology before, during (during recovery from anesthesia), and after stent placement. The CDFI function was used to display major arteries and blood flow across the stent. Spectral Doppler measurements were made to reveal hemodynamic parameters. Vascular enhancement technology (VET) and 3D-TCCS functions were used for image post-processing and to determine stent location, shape, and diameter. All the image taking and spectral Doppler measurements were performed directly online by one experienced sonographer (Yu Wang). The VET image analysis and 3D-TCCS image postprocessing were finished offline by another sonographer (Jian Mei Chen). Both of the interpreters were blinded to the DSA results.

The time point was chosen at pre-stent to access the degree of stenosis, post-stent immediate (in the observation room waiting period right after operation) and post-stent one-week to check for patency of the stent, six-month and two years follow-up to detect restenosis. All the imaging procedures were identical to those described above.

### Statistical Analysis

Each set of data was continuously measured at least three times. The highest flow rates at the site of stenosis were used for data analysis. The SPSS11.5 statistical package was used for data analysis. Mean±standard were used to report measurements. Variance was analyzed based on the preoperative, perioperative, and postoperative values. *P*<0.05 was qualified as statistical significance.

## Results

### Transcranial color Doppler imaging

**Figure 1:**
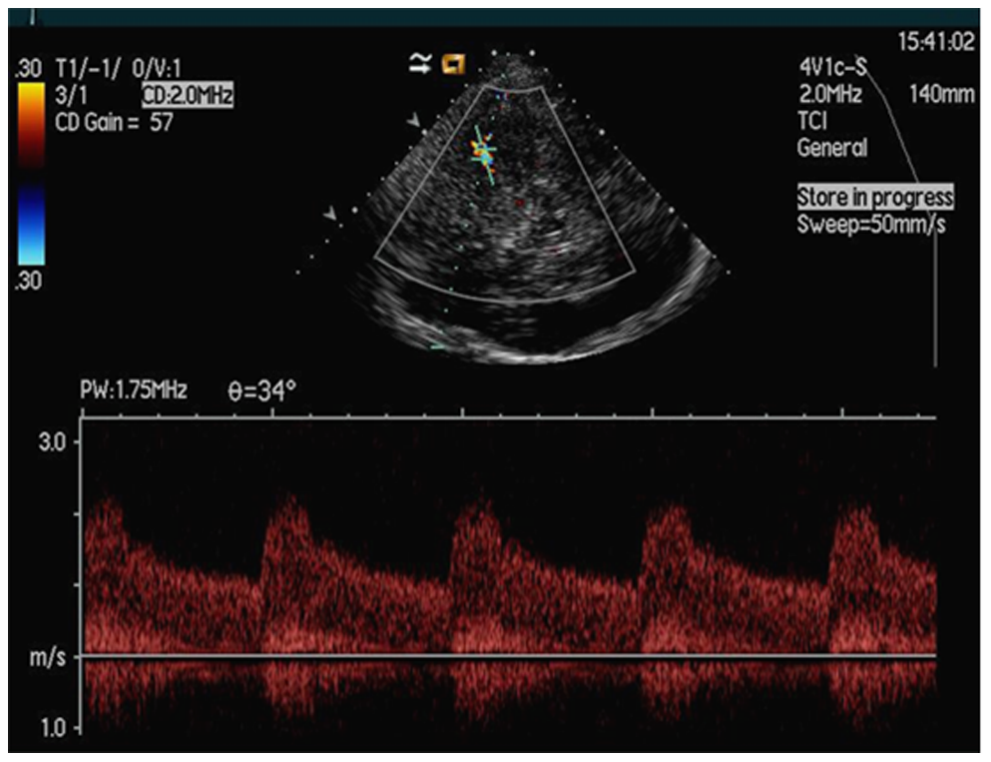
Color Doppler flow imaging (CDFI) revealed bright and mosaic-like color flow of the stenosis before the procedure. Spectral Doppler showed a broadened spectrum with a significantly increased velocity.

**Figure 2:**
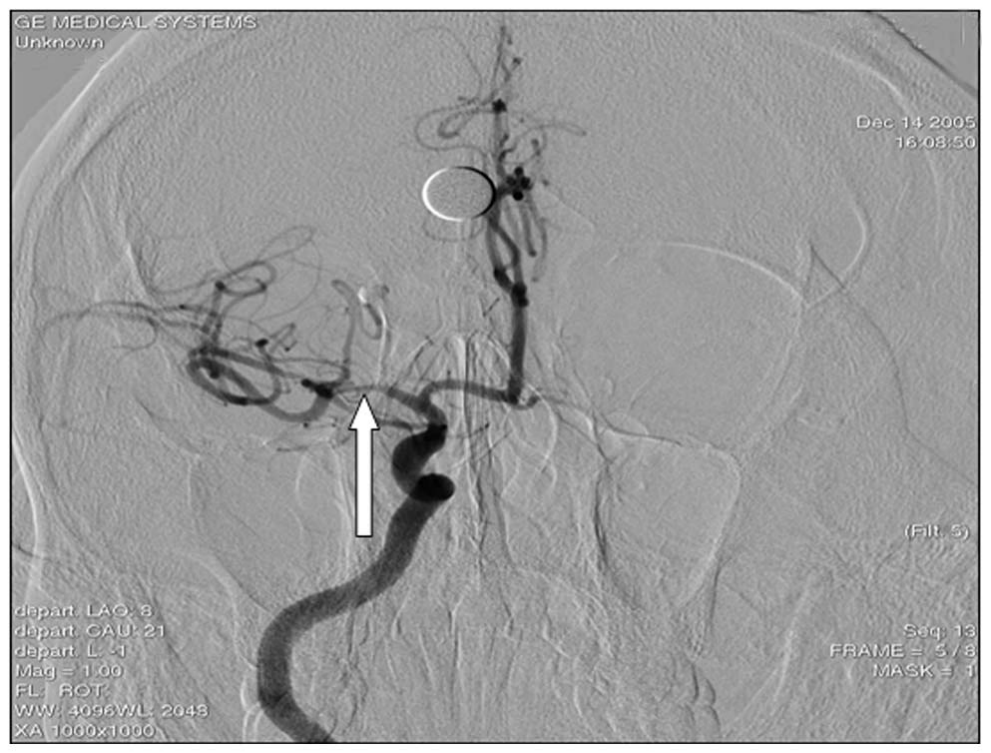
Pre-operative stenosis of MCA indicated by DSA (arrow).

**Figure 3:**
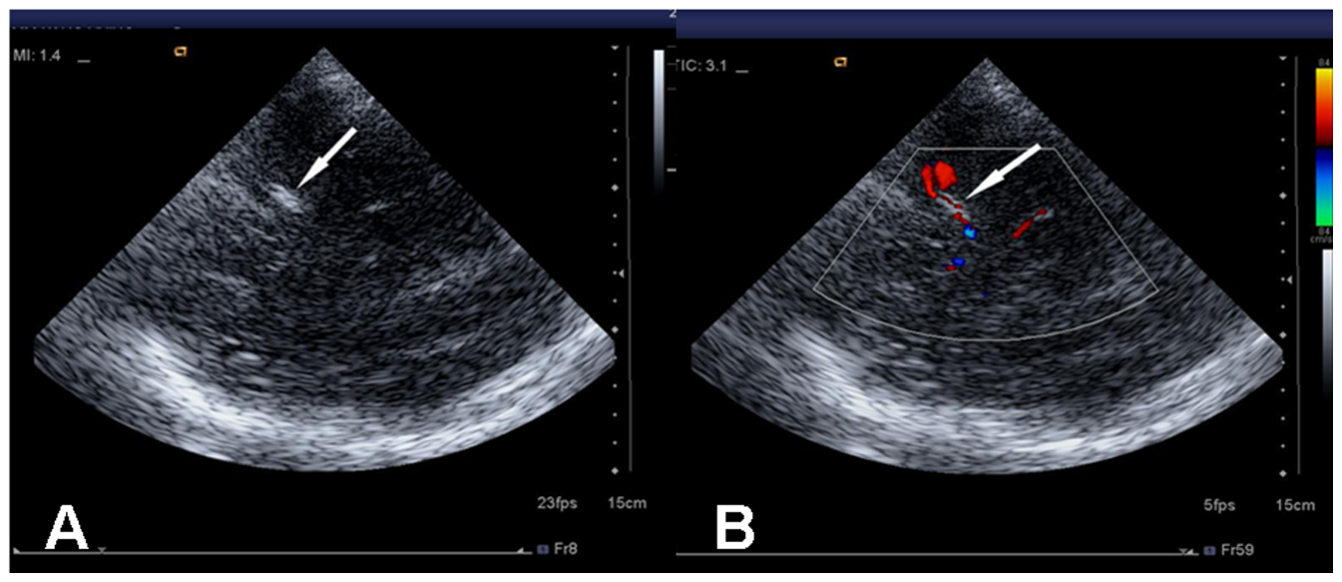
Ultrasonography of post-operative stent. **A**: The stents was shown as hyperechoic images (arrow) on 2D image; **B**: CDFI demonstrated a red bundle of blood flow through the hyperechoic stent (arrow).

**Figure 4:**
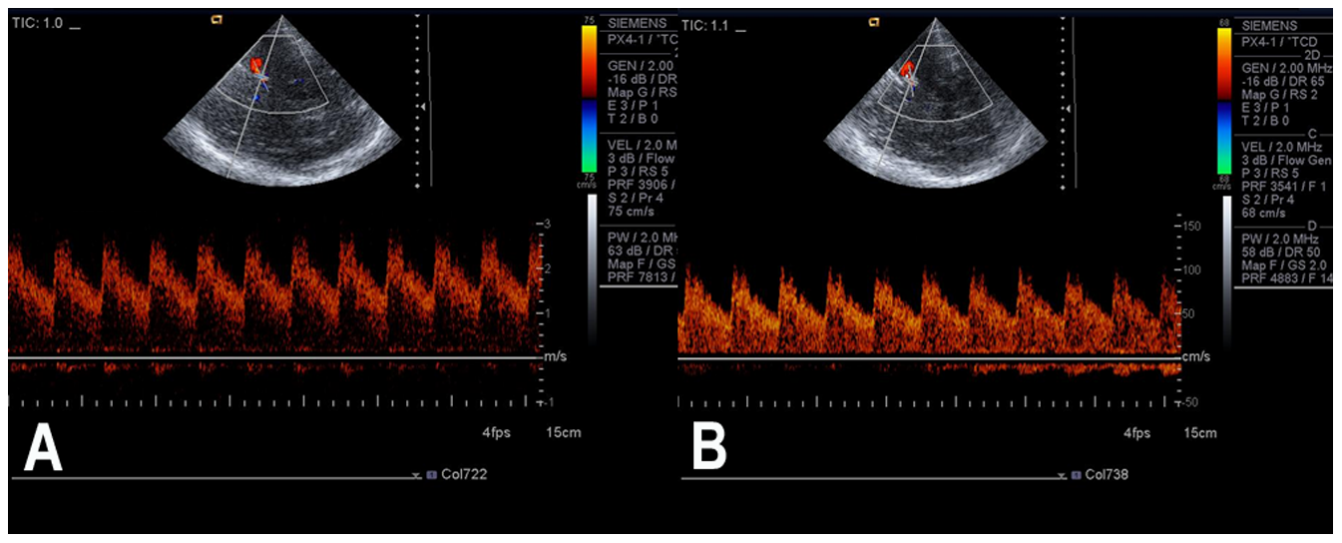
Changes in flow velocity pre- and post- stent placement in one patient. **A,** pre-, Vmax = 280 cm/s; **B,** post-, Vmax = 100 cm/s

2D imaging did not display clear images of the stenosis for all 43 patients pre- angioplasty and stenting. Color Doppler flow imaging (CDFI) revealed bright and mosaic-like color changes in blood flow of the stenotic lesions before the procedure. Spectral Doppler showed a broadened spectrum, suggesting a high impedance spectrum with a significantly increased velocity of blood flow (Figure 1). Audio signals at points of narrowing were low and blunt and showed partial wheeze-like vascular murmurs mixed with high-profile vascular murmur. All the diagnosis was confirmed by DSA before or during the procedure, which demonstrate local diagnosis of M1 segment of MCA (figure 2). Ultrasound was performed in all 43 patients immediately after stent placement (during recovery from anesthesia). Hyperechoic images of the stents, on which stent length can be measured, can be seen in 41 patients out of 43 after the procedure. The stent echo cannot be clearly determined in the remaining 2 patients. CDFI demonstrated the disappearance of the original multicolored mosaic-like blood flow patterns while revealing a bright red bundle of blood flow through the hyperechoic stent (figure 3). When comparing preoperative levels, the Vmax, Vmin, and Vmean at perioperative and postoperative examinations were significantly lower (*P*<0.05) in 42 cases (figure 4). However, perioperative blood flow velocity was a little bit increased in one patient (preoperative flow rate is 223 cm/s and perioperative flow rate is 231 cm/s). Upon CDFI reexamination one week after the procedure, normal blood flow velocity was restored and blood flow was significantly decreased (*P*<0.05) compared to preoperative and perioperative values.

In certain patients, no significant changes in blood flow velocity were found at six-month and two-year follow-up when compared with values at one week after stent placement (*P*>0.05). Doppler measurements before and after stent placement for all patients are shown in table 1.

**Table 1:** Velocity changes of MCA stenosis pre- and post-stent placement (*x*±*s*).

Timing	Vmax (cm/s)	Vmin (cm/s)	Vmean (cm/s)	RI
Pre-stent (n = 43)	282.46±57.7	150.95±51.84	220.76±57.42	0.59±0.11
Post-stent immediate(n = 43)	150.03±23.10*	80.65±8.04*	115.32±13.21*	0.55±0.06
Post-stent 1week (n = 43)	109.59±10.36*^△^	52.32±8.99*^△^	75.95±9.93*^△^	0.54±0.07
Post-stent 6months (n = 12)	109.75±12.84	56.42±8.91	75.92±14.25	0.55±0.06
Post-stent 2years (n = 8)	107.23±10.56	59.45±7.84	79.25±19.84	0.55±0.10

Note: * When compared with pre-stent levels, P<0.05; ^△^ When compared with post-stent immediate levels, P<0.05; MCA: middle cerebral artery; Vmax: peak systolic velocity; Vmin: diastolic velocity; Vmean: time-averaged maximum blood flow velocity; RI: resistance index.

### Ultrasound vascular enhancement technology (VET)

**Figure 5:**
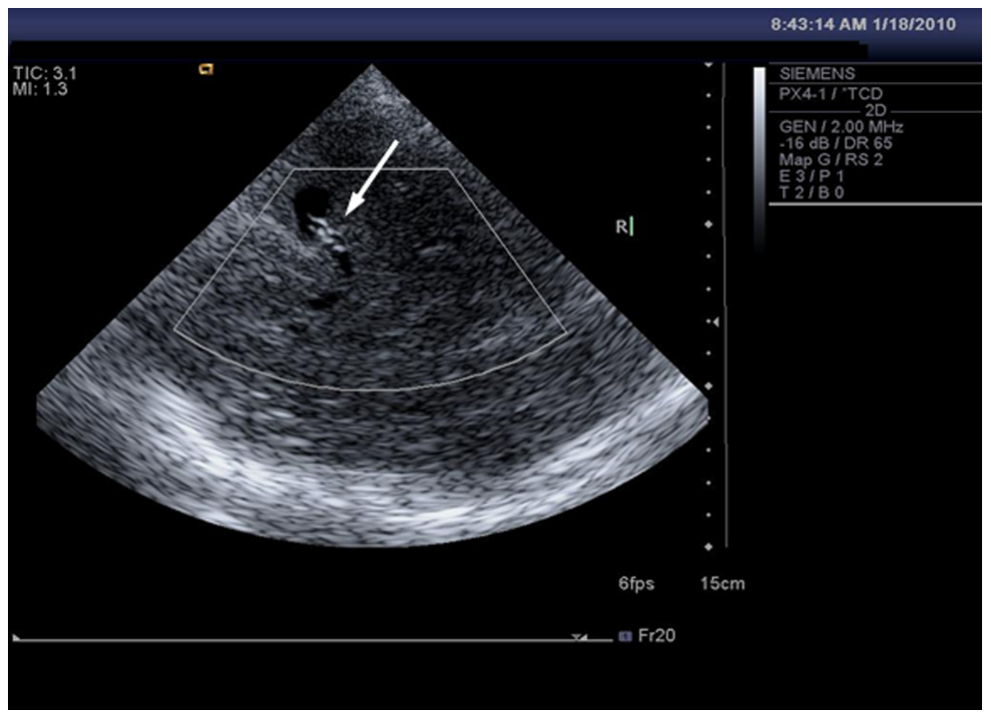
VET imaging showed subtracted blood flow in MCA and hyperechoic stents in the lumen (arrow). This figure comes from the same case of Figure3B.

VET imaging was applied in 32 cases. During preoperative exploration, major intracranial vascular lumen could be clearly visualized. Visible lumen narrowing were seen at the lesion site in stenotic blood vessels, and presented as “corset-like” lesions. In parts of the stenosis, post-narrowing expansions can be seen. Postoperative stent exploration could clearly reveal the lumen and the intraluminal stent position, length, shape, and diameter (Figure 5).

### 3-D Transcranial color-coded sonography (3D-TCCS)

**Figure 6:**
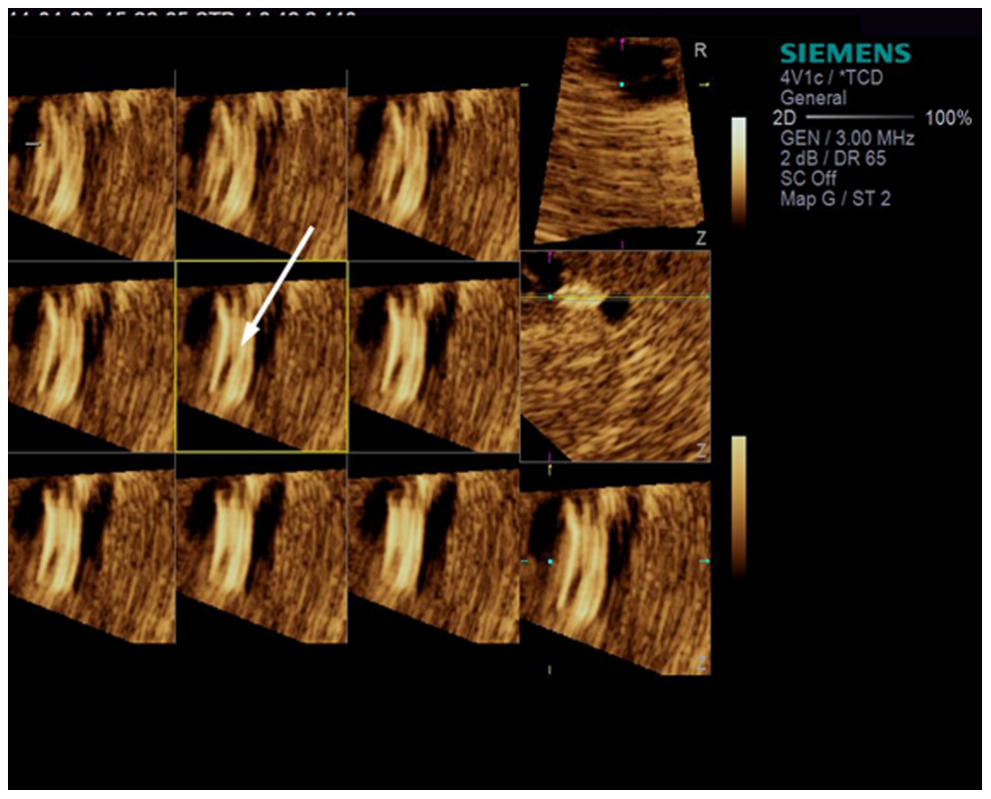
3D-TCCS imaging revealed localized stenosis characterized by hyperechoic signals at the intimal surface (arrow).

We performed 3D-TCCS in 5 patients. Through 3D reconstruction, stent location and vessel wall can be visually displayed. The net-like structure of the stent can be viewed from different angles through reconstruction of 0.2 mm cross-sections. During follow-up examination, 1 patient had increased local blood flow velocity at 178 cm/s. TCCS and VET imaging showed no more detail image on this case. 3D-TCCS imaging revealed localized stenosis characterized by hyperechoic signals at the intimal surface, indicating intimal hyperplasia. The patient was subsequently diagnosed with mild stent restenosis (<50%) with DSA (Figure 6).

## Discussion

In recent years, intracranial vascular stenting is reported as improved therapy for restoring intracranial blood supply and widely recognized. But similar to other endovascular procedures, an effective and convenient method for assessing treatment efficacy and follow-up has not been developed. Insufficient research is available for detection of hemodynamic changes after stent placement; therefore, our group has previously investigated trancranial Doppler ultrasound for diagnosis of intracranial artery stenosis. We have found that, TCCS leads to higher sensitivity, specificity, and accuracy in diagnosing intracranial artery stenosis. Therefore, this technique may be used for the diagnosis and preoperative assessment of cerebrovascular stenosis. In the current study, we enrolled 43 symptomatic patients with MCA stenosis. Through continuous ultrasound imaging, we assessed its clinical value for intracranial artery stenting. Using traditional ultrasound imaging for the hemodynamics assessment of the stenosis, the degree of stenosis was determined mainly based on the velocity of blood flow. Prior to endovascular stenting, DSA was used for diagnostic confirmation in all patients. The assessment of postoperative management is dependent on the parameters recorded through Doppler blood flow velocity measurements. Hemodynamic changes were evaluated perioperatively, for one week after the procedure, and at six-month and two-year follow-up. Based on our results, intravascular stenting led to immediate relief in stenosis accompanied by a dramatic decrease in flow. After one week, due to the reshaping of the stent and vascular remodeling, further improvements in hemodynamics were observed and flow rate continued to decline toward normal levels. At six-month and two-year follow-up, some patients exhibited no significant increases in blood flow velocity, indicating the superior efficacy of stenting. No occurrences of in-stent restenosis were found. In a large population study evaluating MCA implantations, the incidence of in-stent restenosis determined through DSA was 5% [10], indicating that these procedures are safe and efficacious. The follow-up population of this study is relatively small. At six-month and two-year follow-up we did not find any cases of increased blood flow velocity or in-stent restenosis in 20 patients.

Due to the metal composition of the stent, TCCS can clearly display the stent, thereby determining the location and shape of the stent. Color Doppler can be used to assess postoperative blood flow velocity and has indicated that stenting can achieve adequate patency. 2D imaging has shown that TCCS could display in-stent stenosis in 41 patients while lesions in 2 patients were unclear. Factors that may affect this include age, gender, and skull attenuation. However, this study did not show that these patients were female and older in age. Additionally, the location of the stent may also be a factor, where the acoustic window may place further limitations.

In this study, the instantaneous blood flow velocity in one female patient increased after the stenting procedure, which may be affected by her degree of anesthesia and the subsequent recovery. During physical examination, the patient was found to be irritable and hypertensive. The patient's blood pressure was correlated with the cerebrovascular hemodynamics. Hypertension can lead to changes in vascular function and a transient increase in blood pressure may indirectly lead to an elevation in blood flow velocity. Additionally, hyperperfusion syndrome may also be a potential cause, where hemodynamic measurements may indicate increased blood flow velocity in the stented cerebral hemisphere and acute-onset vasospasms may lead to transient abnormal hemodynamics. At one-week follow-up with TCCS, this patient's blood flow velocity had significantly decreased when compared with perioperative values. At six-month follow-up, the maximum blood flow velocity was slightly higher (but within normal limits) than at one-week follow-up with indicating superior efficacy.

The VET technique builds a silhouette image based on blood vessels carrying blood. This technique can provide “reverse enhancement” of microvascular display capabilities and vascular sensitivity, leading to clear images of the vessel lumen and wall structures and significantly reduced volume artifacts [11]. In contrast to 2D imaging, VET can be used to visualize small arteries and intracranial vascular lumina. In the detection of intracranial vascular lesions, VET has advantages over color Doppler imaging on displaying the lumen of vessels because of less frequency reduction. In the study, when compared figure 3B with figure 5 (they come from the same patient), the clarity of TCCS stent lumen images was enhanced with the VET technique, enabling a more objective evaluation of the stent morphology.

Compared with conventional 2D imaging, 3D imaging can provide further detailed information (e.g. multi-angle view of the lesion) such as the threading of stents. For patients with in-stent restenosis, conventional TCCD can detect blood flow acceleration whereas 3D-TCCS can display details of intimal hyperplasia at the stent surface. However, only a few cases in our study utilized 3D imaging, thereby limiting further analysis of its efficacy. Nonetheless, TCCS can be used for the preoperative diagnosis and postoperative follow-up in the treatment of intracranial artery stenosis.

This study is a single-center research. The sample case is limited to accomplish more correlation study to clinical outcomes and comparison study to postoperative DSA or other non-invasive techniques such as CTA and MRA. This is the main limitations of the study. With the accumulation of cases, assessment on reproducibility of the methods would be performed in the future work.

In conclusion, TCCD can be considered a quick and effective clinical detection method to evaluate the stenting treatment for MCA stenosis. Based on the change in hemodynamics, an objective assessment of the clinical efficacy can be performed. New imaging technologies 3D and VET may achieve additional image informations with increased clarity and reliability.

## References

[1] Kim DE, Kim JY, Jeong SW, Cho YJ, Park JM, et al (2012) Association between changes in lipid profiles and progression of symptomatic intracranial atherosclerotic stenosis: a prospective multicenter study. Stroke 43: 1824–1830. 22539545 10.1161/STROKEAHA.112.653659

[2] Schreiber S, Serdaroglu M, Schreiber F, Skalej M, Heinze HJ, et al (2009) Simultaneous occurrence and interaction of hypoperfusion and embolism in a patient with severe middle cerebral artery stenosis. Stroke 40: e478–480. 19498193 10.1161/STROKEAHA.109.549378

[3] Kablak-Ziembicka A, Przewlocki T, Pieniazek P, Musialek P, Tekieli L, et al (2010) Predictors of cerebral reperfusion injury after carotid stenting: the role of transcranial color-coded Doppler ultrasonography. Endovasc Ther 17: 556–631. 10.1583/09-2980.1 20681776

[4] Park S, Lee DG, Chung WJ, Lee DH, Suh DC (2013) Long-term Outcomes of Drug-eluting Stents in Symptomatic Intracranial Stenosis. Neurointervention 8: 9–14. 23515851 10.5469/neuroint.2013.8.1.9 PMC3601283

[5] Qu H, Li J, Zhao X (2013) Factors affecting pre- and post-stenting computed tomography perfusion in patients with middle cerebral artery stenosis. Exp Ther Med 5: 471–474. 23404087 10.3892/etm.2012.805 PMC3570112

[6] Shi MC, Wang SC, Zhou HW, Xing YQ, Cheng YH, et al (2012) Compensatory remodeling in symptomatic middle cerebral artery atherosclerotic stenosis: a high-resolution MRI and microemboli monitoring study. Neurolres 34: 153–158. 10.1179/1743132811Y.0000000065 22334055

[7] Turek G, Kochanowicz J, Rutkowski R, Krejza J, Lyson T, et al (2012) Accuracy of transcranial colour-coded sonography in the diagnosis of anterior cerebral artery vasospasm. Neurol Neurochir Pol 46: 233–238. 22773509 10.5114/ninp.2012.29131

[8] Wong KS, Li H, Lam WW, Chan YL, Kay R (2002) Progression of middle cerebral artery occlusive disease and its relationship with further vascular events after stroke. Stroke 33: 532–536. 11823665 10.1161/hs0202.102602

[9] Moreira T, Michel P, Binaghi S, Hirt L (2012) Risk factor impact on blood flow velocities and clinical outcomes of stented cervical and intracranial stenoses: preliminary observations. Clin Neurol Neurosurg 114: 922–929. 22402202 10.1016/j.clineuro.2012.02.005

[10] Zhang L, Huang Q, Zhang Y, Deng B, Liu J, et al (2013) Single-center study of Wingspan stents for symptomatic atherosclerotic stenosis of the middle cerebral artery. J Clin Neurosci 20: 362–366. 23228656 10.1016/j.jocn.2012.03.033

[11] Liu X, Duan YY, Wang J, Sun SG, Li J, et al (2009) In vitro model test and preliminary clinical application of a new method of ultrasonographic imaging: vascular enhancement technology. Ultrasound Med Biol 35: 1502–1509. 19632758 10.1016/j.ultrasmedbio.2009.04.005

